# Application of Fisetin to the Quantitation of Serum Albumin

**DOI:** 10.3390/jcm9020459

**Published:** 2020-02-07

**Authors:** Jung-Min Park, Van Quan Do, Yoon-Seok Seo, Men Thi Hoai Duong, Hee-Chul Ahn, Hee Jin Huh, Moo-Yeol Lee

**Affiliations:** 1 College of Pharmacy, Dongguk University, Goyang-si, Gyeonggi-do 10326, Korea; 2 Department of Laboratory Medicine, Dongguk University Ilsan Hospital, Goyang-si, Gyeonggi-do 10326, Korea

**Keywords:** fisetin, human serum albumin, albumin assay, clinical laboratory test

## Abstract

Fisetin (3,3′,4′,7-tetrahydroxyflavone) is a widely distributed natural flavonol. It interacts with albumin, and thereby generates a fluorescence signal quantitatively. Based on such optical characteristics, we postulated that fisetin was applicable to the quantitation of albumin as an indicator. To establish the fisetin-based albumin assay, we examined the optical properties of fisetin and fisetin–albumin complex. The assay conditions were fine-tuned to fit for the actual concentration of serum albumin and to generate an optimal signal with a high signal-to-background ratio. The reaction between fisetin and albumin was linear in a wide range of concentrations. Non-protein serum components did not interfere with the reaction. The reactivity of fisetin was apparently specific for albumin among serum proteins. Both plasma and serum were compatible with the assay. The samples could be stored in a refrigerator or a freezer without the loss of reactivity toward fisetin. The generation and decay rates of the signal were acceptable for manual handling. The recovery of fortified albumin in serum was confirmed and the assay was validated with human sera. Fisetin-based albumin assay is suitable for clinical laboratory testing, considering the simple and short procedure, high specificity and sensitivity, linearity over a wide range of albumin concentrations, and, presumably, potential automatability.

## 1. Introduction

Albumins are simple, water-soluble proteins present in body fluids and tissues. They are most commonly found in blood plasma, in which they are often referred to as serum albumin. Human serum albumin (HSA) is synthesized in and secreted from the liver. It is the most abundant plasma protein. Its concentration is typically 35–50 g/L (= 3.5–5.0 g/dL). It plays crucial physiological roles in the maintenance of plasma osmotic pressure, regulation of vascular permeability, and the transport of substances including cholesterol, fatty acids, hormones, and other small molecules [1,2]. Regardless of its functions, HSA is measured to monitor the status of health conditions. Low serum albumin, usually < 35 g/L, can be caused by malnutrition or wasting syndrome [3]. More importantly, such hypoalbuminemia is indicative of various liver diseases [4] and kidney disorders [5]. Accordingly, the serum albumin test is a part of liver and renal panels and a comprehensive metabolic panel [6]. On the other hand, there can be a temporary increase in serum albumin level, which is generally attributed to abrupt dehydration or a high protein diet [7].

The reference methods for serum albumin measurement are immunochemical tests linked to nephelometry or turbidimetry [8]. They are relatively specific, but are time-consuming and less cost-effective. As a result, they are not routinely used for early diagnosis. Alternatively, dye-binding methods using bromocresol green (BCG) and bromocresol purple (BCP) came into wide use because of their relative convenience and cost-efficiency [9]. Despite being widely adopted in clinical laboratories, they are not without disadvantages. BCG is relatively less specific to albumin and reacts with other serum proteins, such as globulins, frequently resulting in overestimation [10]. BCP is comparatively specific to albumin, but it has a lower molar extinction coefficient with bound forms of albumin than BCG. Therefore, the BCP method is less accurate and tends to underestimate albumin in certain abnormal conditions, such as the increase in bilirubin-bound albumin [11]. Indeed, improved methods are under development to overcome the limitations of the current methods [12,13,14].

Fisetin (3,3′,4′,7-tetrahydroxyflavone) is a widely distributed natural flavonol found in plants, including fruits and vegetables. It was studied for biological functions, such as neurotrophic, anti-inflammatory, and other health-beneficial effects [15]. Initially, the interaction of fisetin with serum albumin was reported from the pharmacokinetic study of flavonols [16]. Albumin was found to be a carrier of fisetin in the blood, and the binding of fisetin to albumin was observed to change the intrinsic fluorescence of fisetin [16,17]. Subsequent studies have characterized the interaction between fisetin and albumin, including the conditions affecting interaction, kinetics of reaction, and potential binding sites [17,18]. Based on these studies, we hypothesized that fisetin is applicable to the quantitation of albumin in biological samples. To establish the fisetin-based albumin assay, we examined the optical properties of fisetin and fisetin–HSA complex, optimized the reaction condition, and tested the specificity of fisetin for albumin and the stability of the fluorescence signal. We additionally validated this novel assay with the standard addition method and human sera.

## 2. Materials and Methods

### 2.1. Reagents

Fisetin, HSA, ethylenediaminetetraacetic acid (EDTA), sodium citrate, heparin, γ-globulin, warfarin, ibuprofen, and digitoxin were purchased from Sigma-Aldrich (St. Louis, MO, USA). The TITAN GEL serum protein system was acquired from Helena Laboratories (Beaumont, TX, USA). All other chemicals were purchased from standard suppliers and were of the highest purity available.

### 2.2. Fluorescence Spectra of Fisetin and Fisetin–HSA Complex

A fisetin solution of 30 μM was prepared with phosphate-buffered saline (PBS; 154 mM NaCl, 5.6 mM Na_2_HPO_4_, 1 mM KH_2_PO_4_, pH 7.4) or borate buffer solution (BBS; 0.1 M Na_2_B_4_O_7_, adjusted to pH 9.0 using 0.1 M H_3_BO_3_) with or without 5 mg/mL HSA. The absorbance was measured at 250–500 nm with 1-nm steps using a FP-8200 fluorescence spectrophotometer (Jasco, Easton, MD, USA). The emission was scanned from 400 to 600 nm at 400 nm excitation. The sensitivity of the instrument was arbitrarily set depending on fluorescence strength. To capture a fluorescence image, 30 μM fisetin solutions containing 0–10 mg/mL HSA were put into the wells of a 96-well plate and were excited at 400 nm. An image was acquired with a ChemiDoc XRS+ system and Image Lab software (Bio-Rad Laboratories, Hercules, CA, USA).

### 2.3. Titration Curves of Fisetin and HSA

The titration curves were prepared with 0–100 mg/mL HSA and 1–30 μM fisetin. The reaction was induced by adding 10 μL HSA in distilled water to 90 μL fisetin in BBS. The fluorescence was measured at 400/560 nm excitation/emission wavelengths with a SpectraMax M3 microplate reader (Molecular Devices, Sunnyvale, CA, USA).

### 2.4. Reaction Rate, Signal Stability, and the Impact of Sample Storage Condition and Anticoagulants

The fluorescence was measured at 0.5–10 min after adding 10 μL of 5 mg/mL HSA to 90 μL of 30 μM fisetin to assess the time required for reaction. Similarly, the fluorescence was measured at 30 and 60 min after mixing fisetin and HSA to test the stability of the fluorescence. The impact of HSA storage condition on the reactivity to fisetin was examined by comparing HSA solutions kept at 4 °C or −80 °C for 24 h with a freshly prepared solution. To test the compatibility of anticoagulants with the fisetin-based albumin assay, the HSA solution was prepared to contain acid-citrate-dextrose (ACD; 22 g/L trisodium citrate, 8 g/L citric acid and 24.5 g/L glucose), 3.2% trisodium citrate, 1.8 mg/mL EDTA, or 15 U/mL heparin. Each solution of 10 μL was added to 90 μL of 30 μM fisetin, and the fluorescence intensity was measured using a SpectraMax M3 microplate reader.

### 2.5. Preparation of Human Serum

The human serum samples, after clinical examination, were used regardless of age and illness of donors in Dongguk University Ilsan Hospital (Goyang, Korea). Whole blood was centrifuged at 2700× *g* for 10 min, and the supernatant was taken for experiments. The study was approved by the Institutional Review Board of Dongguk University Ilsan Hospital ethics committee in accordance with the Declaration of Helsinki.

### 2.6. Specificity of Fisetin for Albumin in Serum

The specificity of fisetin for albumin was examined using the TITAN GEL serum protein system. Human sera (3 μL) from five donors were placed on gels. After electrophoresis, the gels were stained with amido black, according to the manufacturer’s instructions, or fisetin by being incubated in BBS containing 30 μM fisetin for 5 min. Colorimetric and fluorescence images were acquired with a ChemiDOC XRS+ system equipped with Image Lab software. The effect of non-protein constituents on the assay was examined with serum filtrate. Serum was filtered by centrifugation at 5000× *g* for 30 min using a Microsep advance centrifugal device with a 3-kDa molecular weight cut-off (Pall Life Sciences, Port Washington, NY, USA). The HSA solution was prepared either in BBS or serum filtrate. These solutions were reacted with 30 μM fisetin, and fluorescence was measured with a FP-8200 fluorescence spectrophotometer.

### 2.7. Thermodynamic Properties of the Reaction

The thermodynamic properties were examined by isothermal titration calorimetry (ITC) using Nano ITC (TA Instruments, New Castle, DE, USA) at 30 °C. The HSA was dialyzed against an ITC buffer solution containing 50 mM phosphate and 1% dimethyl sulfoxide at pH 7.4. Sample cells were filled with 165 μL of 50 μM HSA, and 5 μL of 0.5 mM fisetin was automatically titrated 20 times. The titration intervals were 300 s to reach complete equilibrium. The result was analyzed using NanoAnalyze software 3.8.0 (TA Instruments). The binding ratio (*N*), the enthalpy changes upon binding (Δ*H*), and the dissociation constant (*K_d_*) fit with the independent binding model. The uncertainties of *N*, Δ*H*, and *K_d_* were statistically estimated from 1000 trials of the best-fitting with a 95% confidence interval. The change in binding free energy (Δ*G*) and the change in entropy (Δ*S*) were calculated from the values of Δ*H* and *K_d_*. The binding of warfarin or ibuprofen to HSA was analyzed following the same procedure.

### 2.8. Site Marker Displacement Study

The binding site for fisetin was identified using the site-specific albumin-binding drugs warfarin, ibuprofen, and digitoxin. A decrease in fluorescence of 5 mg/mL HSA and 30 µM fisetin was examined in the presence of 0–500 µM of these drugs.

### 2.9. Recovery of HSA in Fortified Serum Analysis

Four human sera were fortified with 1 or 5 mg/mL HSA. Before and after spiking, albumin was quantified using the standard curve prepared with HSA. The recovery of added HSA was calculated by comparing the albumin concentrations in unspiked and spiked sera.

### 2.10. Validation of Fisetin-Based Assay

The albumin in human sera from 100 donors was quantified with fisetin- and BCG-based assays. For the fisetin method, 10 μL of ten-fold diluted serum was added to 90 μL of 30 μM fisetin solution, and the fluorescence was measured with an FP-8200 fluorescence spectrophotometer. The Cobas c702 automated chemistry analyzer (Roche Diagnostics, Mannheim, Germany) and albumin Gen.2 reagent were used for the BCG-based analysis.

### 2.11. Statistical Analyses

Means and standard errors of means were calculated. To compare two regressions and to determine the significances of the differences, the slopes and Y-intercepts were subjected to Student’s *t*-test. The statistical analyses used SigmaPlot software ver. 13 (Systat Software, San Jose, CA, USA), and *p* < 0.05 was considered statistically significant.

## 3. Results

### 3.1. Fluorescence Spectra of Fisetin and Fisetin–HSA Complex

**Figure 1:**
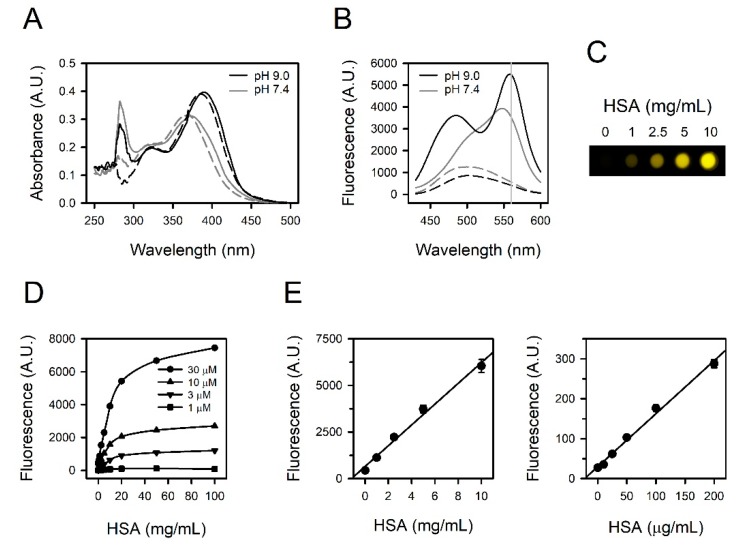
Fluorescence spectra and titration curves obtained from fisetin and HSA. (**A**) The absorbance spectra and (**B**) the emission spectra from excitation at 400 nm were obtained from 30 μM fisetin at pH 7.4 (gray lines) or pH 9.0 (black lines) in the presence (solid lines) or absence (dashed lines) of 5 mg/mL HSA. A gray vertical reference line was drawn at 560 nm (**B**). (**C**) The fluorescence image of HSA-containing fisetin solutions was obtained at excitation at 400 nm. (**D**) The titration curves were acquired with 0–30 μM fisetin and 0–100 mg/mL HSA. (**E)** The standard curves were prepared with 30 μM fisetin and 0–10 mg/mL HSA (left panel) or 1 μM fisetin and 0–200 μg/mL HSA (right panel). The values are presented as means ± standard errors (*n* = 3).

We analyzed the absorbance spectra of fisetin in the absence or presence of HSA. Fisetin was solubilized in PBS at pH 7.4 with or without HSA. The maximal absorbance of fisetin was observed at 360–380 nm (Figure 1A, gray lines). A peak at around 280 nm appeared to be from HSA, as shown in Figure S1 and described previously [18,19]. Considering the previous report that the binding constant increased with the rise of pH over 2.0–9.0 [17], we tested the absorbance in BBS at pH 9.0. The spectra shifted to the right, with maximal absorbance at 380–400 nm (Figure 1A, black lines). The presence of HSA remarkably intensified emission (Figure 1B). The maximal emission was observed at 550−570 nm. The ratio of signals, fisetin–HSA/fisetin was approximately 6.3 (= 3528/563) at pH 7.4 and it was increased up to 13.3 (= 5476/411) at pH 9.0 (Figure 1B, gray vertical reference line). Accordingly, subsequent experiments were conducted using BBS for a better signal-to-background ratio, and the fisetin-based albumin assay was established with BBS. The fluorescence was seemingly in proportion with the concentration of HSA (Figure 1C).

### 3.2. Titration Curves Obtained from Fisetin and HSA

We examined the quantitative relationship of the reaction between fisetin and HSA with 1–30 μM fisetin and 0.1–100 mg/mL HSA. The fluorescence intensity was linearly proportional to the concentrations of both fisetin and HSA up to 30 μM and 10 mg/mL, respectively. It appeared to be saturated above 10 mg/mL of HSA (Figure 1D), and higher than 30 μM of fisetin did not generate a larger signal (data not shown). Accordingly, human serum needs to be diluted approximately 10-fold in the actual assay to cover the albumin concentration in human serum, several tens mg/mL. The fisetin was set to be 30 μM to quantify 0−10 mg/mL HSA (Figure 1E, left panel, r^2^ = 0.9900), and 1 μM fisetin was sufficient for a linear standard curve at 0–200 μg/mL HSA (Figure 1E, right panel, r^2^ = 0.9929), corresponding to the urine albumin level in microalbuminuria (MAU) [20].

### 3.3. Reaction Rate and the Stability of Signals

**Figure 2:**
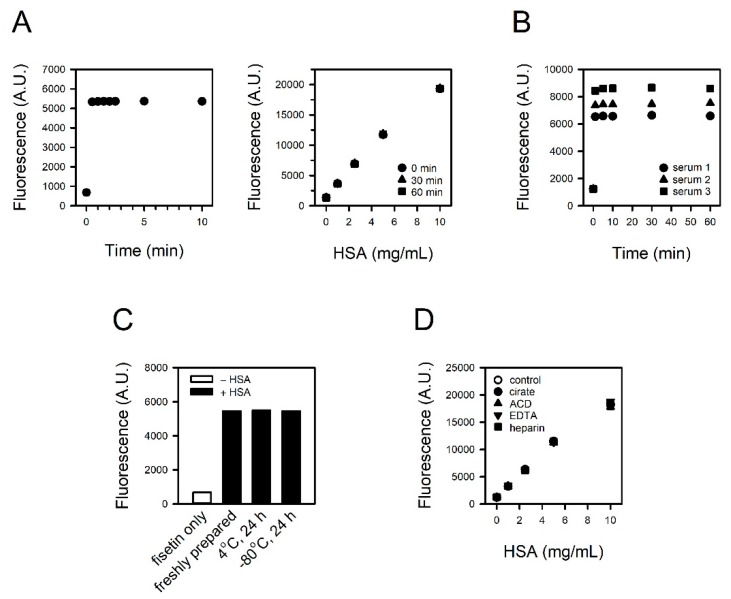
Reaction rate, signal stability, and the impact of sample storage condition and anticoagulants on assay. (**A**) The fluorescence was measured from 0.5 to 10 min after inducing the reaction of 30 μM fisetin and 5 mg/mL HSA (left panel). The stability of the fluorescence signal was tested up to 60 min (right panel). (**B**) The fluorescence from 30 μM fisetin and 10% serum was measured from 1 to 60 min. (**C**,**D**) The HSA solutions were prepared (**C**) freshly or kept at 4 °C or −80 °C for 24 h, and (**D**) to contain citrate, ACD, EDTA or heparin. They were reacted with fisetin, and the fluorescence intensities were measured.

We examined the time required for the reaction and the stability of the fluorescence. The reaction was induced by mixing fisetin and HSA. Stable fluorescence could be detected within 0.5 min (Figure 2A, left panel), and this signal was not altered, at least up to 60 min (Figure 2A, right panel). The reaction-generating fluorescence was sufficiently rapid enough to skip the incubation process, and the decay of the fluorescence seemed relevant for a practical assay. Quite the same results were obtained with three human sera, in addition to HSA solution (Figure 2B).

### 3.4. Impact of Sample Storage Condition and Anticoagulants on the Reaction

We compared refrigerated or frozen HSA solution with freshly prepared HSA solution to test the sample storability. Prior to assay, 5 mg/mL HSA solutions were kept at 4 °C or −80 °C for 24 h and then reacted with 30 μM fisetin. The fluorescence intensities were identical regardless of storage conditions (Figure 2C).

To test the compatibility of anticoagulants with the assay, HSA solution containing citrate, ACD, EDTA, or heparin was reacted with 30 μM fisetin. The presence of anticoagulants did not affect the fluorescence intensity from fisetin–HSA complex (Figure 2D).

### 3.5. Specificity of Fisetin to HSA

**Figure 3:**
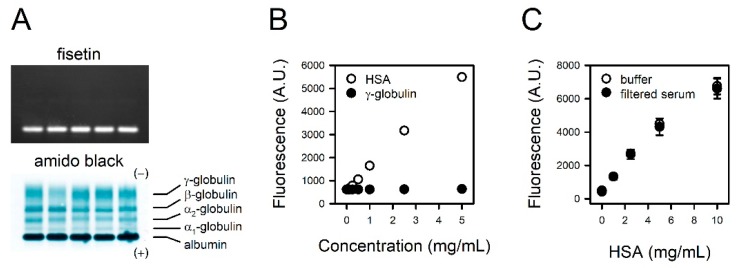
Specificity of fisetin to HSA. (**A**) The proteins in human sera from five donors were separated on agarose gel. The gels were stained with fisetin (upper panel) or amido black (lower panel). (**B**) The HSA or γ-globulin solutions were reacted with fisetin, and the fluorescence was measured. (**C**) The serum was filtered with 3 kDa cut-off filter. Titration curves were obtained with fisetin and HSA solubilized in BBS or serum filtrate. The values are presented as means ± standard errors (*n* = 3).

We examined the specificity of fisetin to albumin through the exclusive staining of albumin in serum. The proteins in human sera from five donors were separated on agarose gel and stained with fisetin or amido black. Typical electrophoretic mobility was observed in the amido black staining; the fastest-moving band, and normally the most prominent, is the albumin band found closest to the anodic edge of the gel. The adjacent faint band is α_1_ globulin, followed by α_2_-, β-, and γ-globulins (Figure 3A, lower panel). In contrast, only albumin was detected in the fisetin staining (Figure 3A, upper panel), indicating its specificity to albumin. For further confirmation, γ-globulin, the second most abundant protein in serum, was reacted with fisetin at the same concentration as HSA. Unlike HSA, γ-globulin did not generate a fluorescence signal up to 5 mg/mL (Figure 3B).

To examine the potential interference of non-protein serum components with the reaction between fisetin and albumin, we prepared HSA standards solubilized in BBS or serum filtrate. The binding curve obtained with HSA solubilized in serum filtrate was identical to that of HSA in BBS (Figure 3C), suggesting that non-protein components in serum do not interfere with the assay.

### 3.6. Binding Properties of Fisetin and HSA

**Figure 4:**
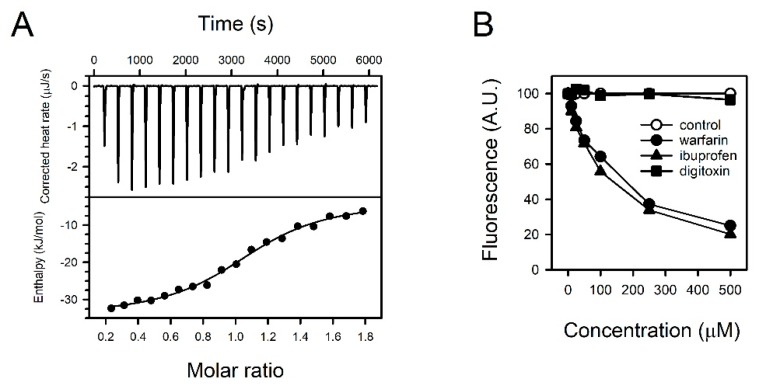
Binding properties of fisetin and HSA. (**A**) The titration curve was prepared with fisetin and HSA employing isothermal titration calorimetry (ITC). The heat rate upon binding was measured (upper panel), and the enthalpy was fitted versus the molar ratio with an independent binding model (lower panel). (**B**) The fisetin in fisetin–HSA complex was displaced by supplementing the binding site-specific drugs warfarin, ibuprofen, or digitoxin. The fluorescence from fisetin–HSA complex without drugs was regarded as 100%, and the relative intensities of fluorescence were presented.

We analyzed the thermodynamics of the interaction between fisetin and HSA by ITC. The binding of fisetin to HSA was exothermic at 30 °C (Figure 4A and Table S1). The heat released upon binding was well fitted to a simple, independent binding model (Figure 4A). ΔH was estimated to be −30.77 ± 3.73 kJ/mol. N of fisetin to HSA and K_d_ were 1.09 ± 0.07 and 5.94 ± 2.63 μM, respectively.

The fisetin did not react with heat-denatured HSA, implying that a tertiary structure was the determinant of fisetin binding (Figure S2). The preferential binding site in HSA was identified with pharmacological tools. The reduction of fluorescence by competition was tested with warfarin, ibuprofen, and digitoxin, which typically bind to albumin binding sites I, II, and III, respectively. Among them, warfarin and ibuprofen attenuated the fluorescence from fisetin and HSA concentration-dependently in a range between 10–500 μM (Figure 4B). Fisetin might bind to sites I and/or II.

### 3.7. Recovery of Fortified Albumin and Quantitation of Albumin in Human Sera

The recoveries of the spiked albumin were calculated to be 105.3 ± 1.4% and 104.9 ± 2.2% for the addition of 1 and 5 mg/mL HSA, respectively (Table 1), suggesting that the fisetin method quantified fortified albumin successfully.

**Figure 5:**
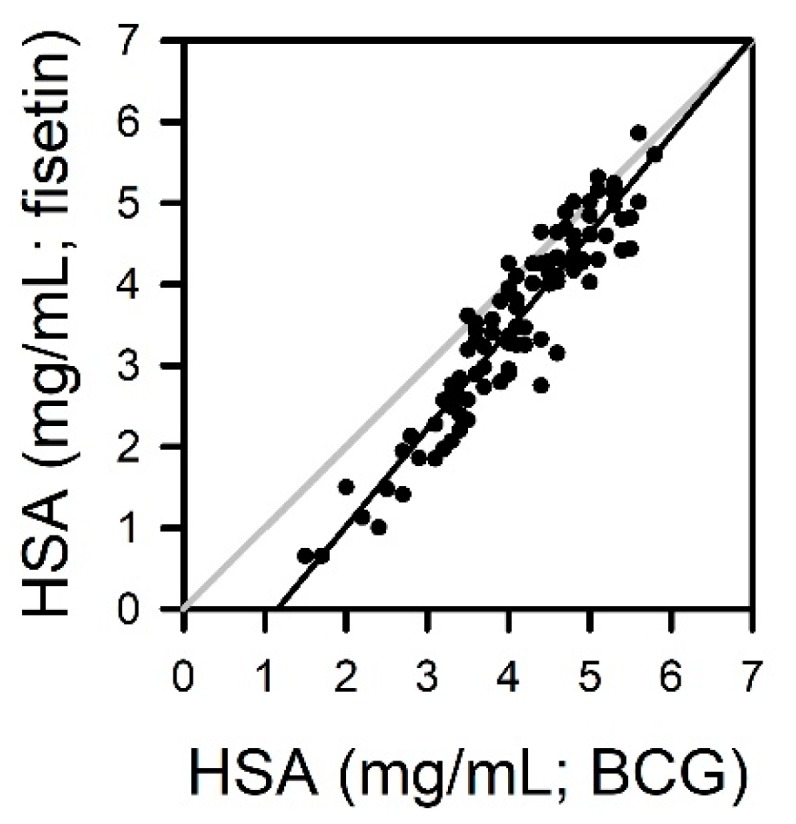
Quantitation of albumin in human sera from 100 donors using fisetin and BCG methods. The albumin concentrations were calculated from the calibration curves obtained with standard HSA. The measurements from the fisetin-based assay were plotted versus values from BCG methods.

The albumin in human sera from 100 donors was analyzed using fisetin and BCG methods. The measured values from the fisetin method and the automated BCG method were plotted on Y- and X-axes, respectively. Means and standard errors of 100 measurements were 3.54 ± 0.12 mg/mL and 4.10 ± 0.09 mg/mL for fisetin and BCG methods, respectively. A slope and Y-intercept were 1.2040 and −1.390 mg/mL, respectively, in linear regression analysis (Figure 5). The measured values from the fisetin-based assay were slightly lower than those from the BCG method, and such a tendency was prominent in the human sera with relatively low albumin.

## 4. Discussion

We investigated the potential application of fisetin to albumin measurements based on the specific binding of fisetin to albumin and its fluorescence-emitting property [16,18]. The assay conditions were fine-tuned to fit the actual concentration range of blood albumin and to generate a high signal-to-background ratio. Typical titration curves could be obtained with fisetin and albumin. The reaction between fisetin and albumin was linear in a wide range of concentrations. As with most chemical reactions, the signal from the reaction reached saturation or plateau at a higher concentration than 10 mg/mL of albumin, presumably due to the limitation of fisetin availability under our experimental condition. Non-protein serum components did not interfere with the reaction. The reactivity of fisetin was apparently specific for albumin among serum proteins. Both blood plasma and serum could be used as samples for the assay. The samples could be stored in a refrigerator or a freezer without any loss of reactivity toward fisetin. The generation and decay rates of the fluorescence signal were not problematic for manual handling or, presumably, instrumental reading. The recovery of fortified albumin in serum was confirmed, and the assay was validated with human sera. In conclusion, fisetin-based albumin assay might be practically applicable to clinical laboratory tests, considering the simple and short procedure, high specificity and sensitivity, linearity over a wide range of albumin concentration, low cost, and potential automatability.

A colorimetric assay using BCG is the most routinely performed albumin assay in clinical laboratories, although immunoassay is generally regarded as the gold standard. It is because the BCG method is easy to run and cost-effective, in spite of certain drawbacks, such as low specificity [10,21]. BCG undergoes a two-step reaction in serum or plasma. It quickly reacts with albumin, which is immediately followed by relatively slow reactions with other components such as globulins [22]. Therefore, an improper reaction time tends to result in the overestimation [10,22,23,24,25]. In the same context, the possibility of diagnostic misinterpretation increases if albumin decreases and globulin increases, such as in nephrotic syndrome [26,27]. On the other hand, fisetin was apparently specific to albumin, and the prolonged reaction did not increase the fluorescence signal up to 60 min (Figure 2 and Figure 3). A fisetin-based albumin assay is specific and free from the error-prone handling procedure.

The fisetin-based albumin assay was validated with random human samples in comparison with the BCG method (Figure 5). The measured values from the BCG method seem to be slightly higher than the results obtained with fisetin (4.10 ± 0.09 mg/mL vs. 3.54 ± 0.12 mg/mL), especially in sera with a low albumin level. The reason for the discrepancy is currently elusive, but it might be attributed to the tendency of BCG to overestimate albumin, as described above. Indeed, quite similar results have been reported in previous studies comparing the BCG method with serum protein electrophoresis [10,21,28]. The mean values of albumin from 166 sera were 4.29 and 3.68 g/dL for the BCG method and electrophoresis, respectively [10]. In the other study, they were 3.76 and 3.13 g/dL in the analysis of 144 sera, respectively [28]. These studies found the overestimation of albumin by the BCG method, especially in sera with low albumin level, but a good agreement of protein electrophoresis with the results from immunoprecipitation methods [10,21]. Further study is needed to explicate the discrepancy between the fisetin and BCG methods.

The signal from fisetin—HSA was linear in a wide range of albumin concentrations, 10 μg/mL–10 mg/mL, which encompasses albumin concentration in MAU. MAU is generally defined as an albumin excretion of 30–300 mg/day or an albumin/creatinine ratio of 20–200 mg/g, which is equivalent to 20–200 mg/L of a spot urine sample [20]. Accordingly, fisetin was expected to be applicable to the measurement of urine albumin. However, it was not feasible, because urine itself had a high autofluorescence (data not shown). Indeed, urine is well-known to have autofluorescence over a wide range of wavelengths, although not all fluorophores in urine were identified [29]. It is also not certain whether the tertiary structure of albumin for fisetin binding can be conserved in harsh conditions, such as extreme pH and ionic strength in urine, although pH issues in the assay can be overcome to some extent by increasing the assay buffer capacity [17,30]. The available methods for diagnosing MAU are limited, as they require high sensitivity because the albumin concentration in urine is very low. Although immunochemical methods are widely used as gold standards, there are fundamental drawbacks ascribing to the quality of antibody and the heterogeneity or fragmentation of albumin in urine. It would be highly valuable if a simple assay method was available for the diagnosis of MAU. Unfortunately, fisetin-based assay is not likely to be applicable to urine albumin due to the autofluorescence of urine.

The fluorescence intensity from fisetin—Albumin complex increased and the emission spectrum in longer wavelengths than 550 nm shifted to the right as the pH increased (Figure 1B). A pH increase causes a structural change in albumin domain I, which may favor the binding of albumin to fisetin [31]. Thus, pH elevation leads to an increase in the binding constant and the affinity for fisetin [17]. It is not clear how pH induces a redshift of emission spectrum. According to the study on quercetin, another flavonol with quite a similar structure to fisetin, pH elevation induces the deprotonation of hydroxyl groups in flavonol structure, and this deprotonation causes the bathochromic shift of the emission spectrum [32]. Indeed, similar deprotonation of hydroxyl groups was detected in fisetin at pH 9.0 [33]. Presumably, the redshift of the fisetin spectrum was attributed to such structural change, depending on the pH.

Fisetin appears to react with HSA at 1:1 ratio (Table S1). Both warfarin and ibuprofen could replace fisetin in fisetin–HSA complex. A straightforward interpretation is that fisetin interacts with both sites I and II, and that the interaction with one site might prevent that of the other site. The preference for site I or II does not seem to differ from each other, considering the similar *K_d_* values of warfarin and ibuprofen to HSA (Figure S3 and Table S1) and their comparable fisetin-replacing capabilities (Figure 4B). Indeed, the binding pockets in HSA are quite flexible and there can be an allosteric interaction among the sites, although the characteristics of sites I and II toward ligands are different [34,35]. Unlike our study, a previous study suggested site I as a major binding pocket for fisetin, based on fluorescence anisotropy and molecular docking studies [17]. This discrepancy remains hard to explain. However, there are reports suggesting the limitations of pharmacological tools for identifying binding sites, owing to limited specificity [36]. Indeed, there are pharmacokinetic studies showing that ibuprofen can displace warfarin or quercetin, another site I-binding molecule, from binding sites [37,38]. More advanced methods will be required for characterizing the binding sites.

## Figures and Tables

**Table 1:** Recovery of HSA in supplemented serum analysis.

	Plasma Albumin (mg/mL)	Added Albumin (mg/mL)	Measured Total Albumin (mg/mL)	Recovery of Added Albumin (%)
serum 1	2.11	1.00	3.29	105.82
		5.00	7.57	106.50
serum 2	2.92	1.00	4.20	107.09
		5.00	8.45	106.68
serum 3	3.03	1.00	4.21	104.40
		5.00	8.41	104.80
serum 4	3.43	1.00	4.61	104.02
		5.00	8.59	101.83

## Supplementary Materials

The following are available online at https://www.mdpi.com/2077-0383/9/2/459/s1, Figure S1: Absorbance spectra of human serum albumin (HSA), Figure S2: Non-reactivity of fisetin to denatured HSA. Figure S3: Titration curves obtained with HSA and (A) warfarin or (B) ibuprofen employing isothermal titration calorimetry (ITC). Table S1: Thermodynamic signatures of fisetin, ibuprofen, and warfarin to HSA.
