# Supplementary material for: Application of Fisetin to the Quantitation of Serum Albumin
Source: J Clin Med. 2020 Feb 7;9(2):459. doi: 10.3390/jcm9020459 (PMC7073753; doi:10.3390/jcm9020459)
Supplement: Supplementary file 1 [file jcm-09-00459-s001.pdf]

Journal of
Clinical Medicine

Supplementary Data

Application of Fisetin to the Quantitation of Serum Albumin

Jung-Min Park [1], Van Quan Do [1], Yoon-Seok Seo [1], Men Thi Hoai Duong [1], Hee-Chul Ahn [1], Hee Jin Huh [2] and Moo-Yeol Lee [1,*]

[1] College of Pharmacy, Dongguk University, Goyang-si, Gyeonggi-do 10326, Korea; vimrifle@naver.com (J.M.P.), dsdoquan@gmail.com (V.Q.D.), tjdbstjrtl11@naver.com (Y.S.S.), hoaimenduong@gmail.com (M.T.H.D.), hcahn@dongguk.edu (H.C.A.)

[2] Department of Laboratory Medicine, Dongguk University Ilsan Hospital, Goyang-si, Gyeonggi-do 10326, Korea; hjhuh@duih.org

* Correspondence: mlee@dongguk.edu; Tel.: +82-31-961-5222

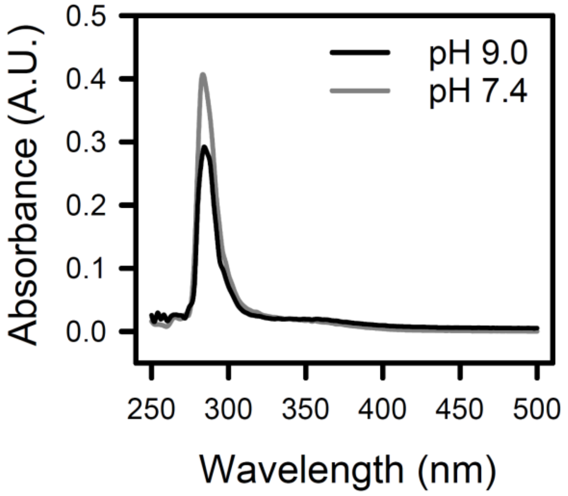

Figure S1. Absorbance spectra of human serum albumin (HSA). HSA was solubilized in phosphate-buffered saline (PBS) at pH 7.4 (gray line) or borate buffer solution (BBS) at pH 9.0 (black line). Absorbance was scanned from 250 to 500 nm using a SpectraMax M3 microplate reader (Molecular Devices).

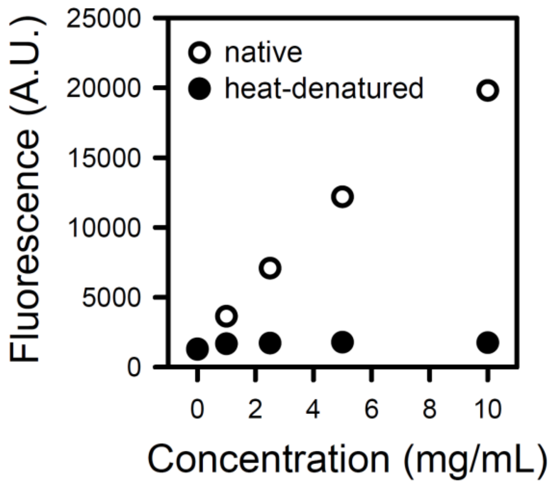

Figure S2. Non-reactivity of fisetin to denatured HSA. HSA was heat-denatured by incubation at 99 ˚C for 15 min. Indicated concentrations of native or denatured HSA were added to 30 μM fisetin. Fluorescence intensity was measured using a SpectraMax M3 microplate reader (Molecular Devices).

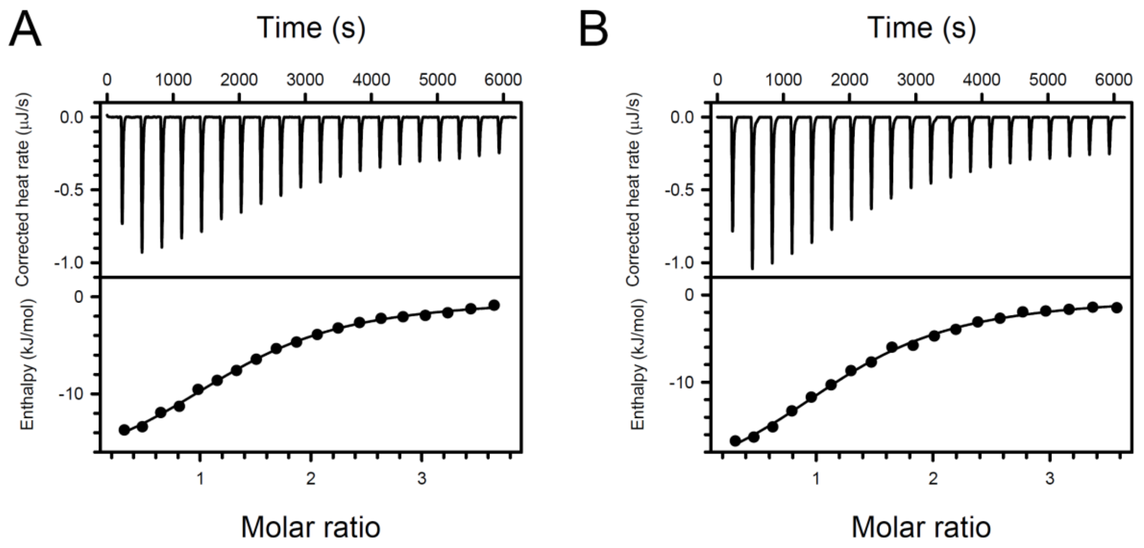

Figure S3. Titration curves obtained with HSA and (A) warfarin or (B) ibuprofen employing isothermal titration calorimetry (ITC). HSA was dialyzed against ITC buffer solution. Sample cells were filled with 165 μL of 50 μM HSA, and 5 μL of 0.5 mM warfarin or ibuprofen were automatically titrated 20 times. Heat rate was measured with Nano ITC (TA Instruments), and the result was analyzed using NanoAnalyze software 3.8.0 (TA Instruments).

Table S1. Thermodynamic signatures of fisetin, ibuprofen, and warfarin to HSA.

	K_d (μM)	ΔH (kJ/mol)	TΔS (kJ/mol)	ΔG (kJ/mol)	N
fisetin	5.94 ± 2.63	−30.77 ± 3.73	−0.44	−30.33	1.09 ± 0.07
warfarin	16.11 ± 5.90	−23.04 ± 3.20	4.78	−27.82	1.30 ± 0.07
ibuprofen	16.27 ± 5.07	−18.71 ± 2.21	9.08	−27.79	1.38 ± 0.06
